# How ADA methodology informs SARS-CoV-2 assay development

**DOI:** 10.4155/bio-2020-0220

**Published:** 2020-09-11

**Authors:** Caroline Becker

**Affiliations:** ^1^Translational Medicine Department, QPS LLC, Delaware Technology Park, 3 Innovation Way, Suite 240, Newark, DE 19711, USA

**Keywords:** antidrug antibody, assay, coronavirus, COVID-19, rRT-PCR, SARS-CoV-2, serology

Of the seven known viruses in the coronavirus family infecting humans, SARS-CoV-2 is emerging as one of the deadliest and most infectious. This novel betacoronavirus is a positive-sense, single-stranded RNA virus that codes for four proteins: the spike protein, the envelope protein, the membrane protein and the nucleocapsid protein [1–3]. SARS-CoV-2’s spike protein binds to a host cell via the ACE2 receptor found on human cells [4,5]. Accurate tests that can help identify active and past SARS-CoV-2 infections are vital during this worldwide pandemic.

## Serology testing

Serology testing can identify antibodies produced when a subject is infected with a virus or other pathogen. IgG antibodies can be reliably detected by a serology test 2–3 weeks after infection [6,7]. Serology testing is not used for diagnosing active infections, but it has other important uses such as identifying past infections, determining potential immunity and screening convalescent plasma. Public health uses include estimating infection rates and the overall fatality rate as well as characterizing transmission rates during contact investigations.

The US FDA has granted an Emergency Use Authorization (EUA) for many serology tests for SARS-CoV-2 [8]. The agency recommends validation of the following:

- Cross-reactivity/analytical specificity to ensure the assay does not react with antibodies of other common viruses;
- Class specificity to ensure that the class of antibody is recorded as labeled;
- Clinical agreement assessing the sensitivity and specificity of the assay.

Performance of a serology assay is primarily based on sensitivity and specificity. Sensitivity, or percent positive agreement, estimates the percent of PCR-confirmed positive samples that are screened positive in the test, which is expected to exceed 90%. Specificity estimates the true negatives that are screened negative, which is expected to exceed 95%.

The established sensitivity and specificity of a serology assay are only estimates and are limited by the samples used to characterize and validate the tests. The more samples of known positive or negative origin used, the narrower the confidence intervals become. Because of the variation among individuals between the onset of the infection and seroconversion, a well-characterized demographic is crucial in understanding the limitations of an assay.

## Anti-drug antibody science & serology assays

Industry experience with anti-drug antibody (ADA) assay development is relevant and can be applied in the design of a specific serology assay. During the development phase, the ADA method and the serological assay method share many similarities:

- Identification of a well-characterized, purified capture antigen;
- Identification of selective detection reagents;
- Use of effective positive and negative controls to assess assay performance;
- Evaluation and identification of the best buffer composition to minimize background without diminishing specific signal;
- Optimization of reagent working concentration, including use of minimum required dilution to mitigate nonspecific binding in the assay.

As with ADA methodology, it is important in the development of a serology assay to set a cut point that distinguishes a positive result from a negative result. During validation of the method, a large sampling of drug-naive (in the case of ADA) or known negative samples (when a comparator method is available) should be utilized to estimate the natural variation of assay signal. The observed variation should be incorporated into selecting or calculating the cut point; the location of the cut point will impact both the estimated sensitivity and specificity.

According to ADA recommendations, an assay that is intended to support safety and efficacy during clinical trials should prioritize minimizing false negatives [9–13]. A multi-tiered approach for detecting potential ADA is recommended, with a screening assay that has a false-positive rate of approximately 5% followed by a confirmatory assay that shows reactivity to the therapeutic protein and targets a false-positive rate of 1%. It is also recommended to perform outlier analysis on the drug data set used to calculate the assay cut point to reduce variation and set a more conservative limit [9–13].

In contrast to ADA methodology, a serology test should prioritize specificity (ideally a false-positive rate of less than 2%), but balance that priority with sensitivity. To ensure maximizing both sensitivity and specificity, it is recommended to evaluate the known negative and positive datasets in combination. When calculating a cut point, all of the negative sample signals in the data set should be considered, without removing outliers. Additionally, a receiver-operator characteristic curve can be used to graph the sensitivity against the inverse of the specificity using all instances of the positive/negative separation cut point in order to target the optimal balance between sensitivity and specificity [14].

## Serology testing development

The QPS team approached developing a serology assay using the spike protein as the capture antigen. We evaluated various recombinant proteins and selected the region in the S1 subunit containing the receptor binding domain antigen as the capture antigen, as it is the most dissimilar to other coronaviruses and contains the region needed to bind the ACE2 protein on the cell’s surface [15,16]. Antibodies targeting the receptor binding domain are most likely to have a neutralizing effect on the virus, making it a specific and highly relevant antigen [15,16].

Removing outliers in different statistical approaches may not affect sensitivity, but it does alter specificity significantly. Part of the reason for the minimal shift in sensitivity with different cut points is that most of the positive population matrix has clear, strong reactivity in the assay. The smaller overlap between the populations results in higher estimates of sensitivity and specificity. By utilizing an approach to set the cut point at the statistically derived 99.9% confidence interval, our team reported a small number of false negatives and minimal false positives. In some cases, the low reactivity for the confirmed positive matrix can be explained by the sampling time.

When calculating the clinical agreement after subgrouping the positive samples, we demonstrated that the sensitivity is influenced by the timing of when a sample is taken. A longer period for seroconversion correlates with increased clinical agreement of positive samples.

- Samples collected 1–13 days after a confirmatory PCR test have a positive agreement of only 70%.
- Samples collected between 14 and 21 days have an 88% positive agreement.
- Samples collected after 21 days have a 97.8% positive agreement.
- Samples were evaluated which were collected as long as 49 days after confirmation of infection and showed no correlation of diminished reactivity over time.

Serology tests are valuable for both individual health and public health purposes when an appropriate cut point is calculated. This type of test is and will remain an important tool in understanding the behavior of SARS-CoV-2 in the body and its infection rate among the population.
